# Adipocyte- and Monocyte-Mediated Vicious Circle of Inflammation and Obesity (Review of Cellular and Molecular Mechanisms)

**DOI:** 10.3390/ijms241512259

**Published:** 2023-07-31

**Authors:** Natalia Todosenko, Olga Khaziakhmatova, Vladimir Malashchenko, Kristina Yurova, Maria Bograya, Maria Beletskaya, Maria Vulf, Larisa Mikhailova, Anastasia Minchenko, Irina Soroko, Igor Khlusov, Larisa Litvinova

**Affiliations:** 1 Center for Immunology and Cellular Biotechnology, Immanuel Kant Baltic Federal University, 236001 Kaliningrad, Russia; tod_89@mail.ru (N.T.); olga_khaziakhmatova@mail.ru (O.K.); vlmalashchenko@kantiana.ru (V.M.); kristina_kofanova@mail.ru (K.Y.); mbograya@mail.ru (M.B.); mariyabel@bk.ru (M.B.); mary-jean@yandex.ru (M.V.); mihalysa@mail.ru (L.M.); minchenkoananastasia@yandex.ru (A.M.); ivs-180@rambler.ru (I.S.); khlusov63@mail.ru (I.K.); 2 Laboratory of Cellular and Microfluidic Technologies, Siberian State Medical University, 2, Moskovskii Trakt, 634050 Tomsk, Russia

**Keywords:** monocytes, adipocyte, metabolic syndrome, obesity, inflammation

## Abstract

Monocytes play a key role in the development of metabolic syndrome, and especially obesity. Given the complex features of their development from progenitor cells, whose regulation is mediated by their interactions with bone marrow adipocytes, the importance of a detailed study of the heterogeneous composition of monocytes at the molecular and systemic levels becomes clear. Research argues for monocytes as indicators of changes in the body’s metabolism and the possibility of developing therapeutic strategies to combat obesity and components of metabolic syndrome based on manipulations of the monocyte compound of the immune response. An in-depth study of the heterogeneity of bone-marrow-derived monocytes and adipocytes could provide answers to many questions about the pathogenesis of obesity and reveal their therapeutic potential.

## 1. Introduction

Obesity is a global human problem that has reached the scale of a worldwide epidemic. Obesity is a leading cause of type 2 diabetes (T2DM), which affects 537 million people worldwide and could reach 784 million by 2045 [1].

The continuum of chronic obesity leading to metabolic disorders (e.g., insulin resistance and T2DM) is of concern to physicians worldwide. The components of metabolic syndrome (MetS) are abnormal growth of adipose tissue through hypertrophy and hyperplasia of fat cells, elevated blood pressure, high blood glucose, and dyslipidemia [2]. 

Obesity is associated with an increase in circulating monocytes [3] and their recruitment to inflamed adipose tissue (AT) in animals and humans [4]. Insulin resistance associated with obesity develops against the background of an increase in the number of tissue macrophages (in adipose, muscle, and liver tissues) and their enhanced pro-inflammatory effects. In addition, increased glycolysis and activation of hypoxic-induced factor 1-alpha (HIF-1α) are observed in macrophages, which stimulate the production of interleukin (IL) 1β and simulate conditions of pseudohypoxia [5]. The pool of tissue macrophages is replenished by infiltration of circulating monocytes [6]. In models of inflammatory diseases, monocyte-derived cells play a pro-inflammatory role by causing tissue damage. In this context, blocking the recruitment of monocytes to inflamed tissues by using nanoparticles or si-RNA against C-C chemokine receptor type 2 (CCR2) is a promising direction of targeted therapy [7] that has only been investigated in animal models.

In general, the role of individual circulating monocyte subpopulations in obesity remains to be elucidated [4]. It is not known when monocytes are activated: before they invade tissues or during differentiation into macrophages in obesity and T2DM. The close relationship between monocytes and adipocytes in obesity is not well understood [8], although [4] highlights the pathogenetically important role of interactions between adipocytes and immune cells in obesity.

Bone marrow adipocytes (BMAs) not only play an important role in regulating the local bone marrow (BM) environment but may also contribute to whole-body homeostasis [9]. Adipocytes can initiate monocyte chemoattractant protein-1-mediated M1 macrophage accumulation in visceral adipose tissue [10]. However, little is known about the potential of BMAs and monocytes to trigger chronic inflammation in obesity and T2DM [11].

The aim of this review is to describe the heterogeneous populations of monocytes and BMAs, along with their role and interaction in myelopoiesis and inflammation in obesity.

## 2. Adipocytes in Bone Marrow

There are at least three widely accepted types of adipocytes (white, brown, and beige or brite (brown/white)), based on their appearance, function, and site of origin [12,13,14]. The bone marrow adipocyte (BMA) may represent a distinct (fourth) type of adipocyte [15]. Both BMAs and beige adipocytes are present in bone marrow. BMAs are thought to be derived from mesenchymal stem/stromal cells (MSCs), often referred to as skeletal stem cells [13,14].

However, it remains controversial whether MSCs are a single source of adipocyte progenitor cells or a number of different progenitor cells [9]. At the very least, adipocyte heterogeneity may also be due to lipid-loaded CD9+, CD45+, and CD55+ macrophages [8]. In turn, bone-marrow-resident macrophages are the central homeostatic element of erythroblastic [16] and erythro-(myelo)-blastic islands [17], and they form the hematopoietic stem cell niche [18].

BMAs are localized in the bone cavity along with hematopoietic cells, trabecular bone, nerve fibers, blood vessels, and sinusoidal capillaries [19] and are a component of the bone marrow stroma [20], which are required for the maturation and proliferation of hematopoietic cells [21], as well as being an important structural and functional element of the hematopoietic inductive microenvironment (HIM) [22].

**Figure 1:**
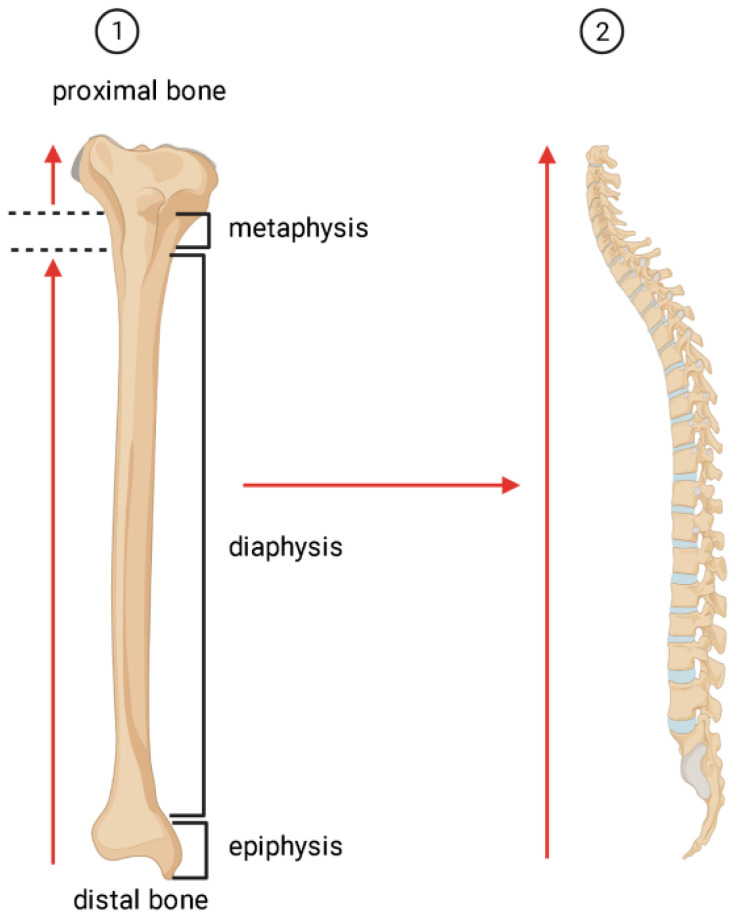
Schematic representation of the direction of adipocyte expansion and yellow bone marrow formation (using the tibia (and spine) as an example). 1—The yellow medulla of the long bones forms at the distal epiphyses, then at the middle diaphyses and fills the medullary canal in adulthood (with the exception of the proximal hematopoietic metaphysis); 2—Then the extension of the yellow marrow affects the spine, starting from the sacral and lumbar region.

According to Neumann’s law [23], after birth, the number of adipose cells in the red (hematopoietic) bone marrow gradually increases, and yellow BM is formed [19], with a decrease in the major proportion of hematopoiesis in the BM cavity of the long tubular bones (Figure 1).

In adults, BMAs comprise approximately 10% of the total mass of adipose tissue [24], and they are large cells (diameter 50–300 μm) [25]. Normally, by age 25, about 70% of the volume of BM is filled with BMAs [24]. In adult men, this indicator is higher in the spine, sacrum, and hips than in women. However, in women over 65 years of age, the fat content in the spine is 10% higher than in men [19,24]. In this case, the adipocytes form the yellow medulla closer to the center of the medullary cavity near the sinusoidal venous capillaries, whereas the red medulla is located in the endosteal zone [26]. Other data suggest that expansion of BMAs occurs in a centripetal pattern: first in the distal skeleton (i.e., lower and upper limbs), then in the epiphyses and diaphyses of the long bones, and later in the capture of the axial skeleton (i.e., cervical, thoracic, lumbar, and sacral spine) [23]. It has been documented that stable yellow BM in the long bones occurs first in the distal epiphyses and originates from the middle diaphyses and fills the medullary canal in adulthood, with the exception of the proximal metaphysis, which remains hematopoietic until old age [23].

BMA tissue has essentially the same properties as WAT. The marker for brown adipose tissue, uncoupling protein (Ucp1), is upregulated in the tibia in response to exercise or in combination with rosiglitazone, resulting in a decrease in BMA tissue volume, size, and number of BMAs [27]. In BMA tissue homeostasis, *Ucp1* gene expression can be detected in the metatarsals, indicating specific heterogeneity and plasticity of BMAs [23]. BMAs are unique metabolically active cells with abundant lipid stores, mitochondria, and endoplasmic reticulum [28]. BMAs are a distinct population of adipocytes that share some of the characteristics of white and brown adipocytes [29,30].

The BMA tissue in the yellow BM of the distal tibia (and the caudal spine in mice) contains adipocytes formed at early developmental stages [31], which are stable, constitutive BMAT (cBMA) tissue [19]. They are large and composed of unsaturated lipids. In humans, cBMA tissue first appears in the terminal limbs of the fetus just before birth; cBMA tissues are located in close proximity to one another, with no intervening hematopoietic cells [32].

cBMA tissue resembles WAT and is more stable than regulated BMA (rBMA) tissue; rBMA tissue is localized in the red CM of the tibia proximal to the junction with the fibula in the axial skeleton. rBMAs occur singly and in groups between hematopoietic cells (in areas of active hematopoiesis) [19]. Labile rBMAs fill the medullary canal just below the growth plate of the primary sponge tissue and appear in the secondary ossification center [9]. They are smaller, contain predominantly saturated lipids, and are readily mobilized upon stimulation (in response to hematopoietic demand) [23].

At the same time, the development of rBMAs differs in the long bones of different mouse lines (C57B1/6J, C3H/HeJ) and is not observed in vertebrae [19].

## 3. Origin/Formation of Bone Marrow Adipocytes

It is still unclear whether one cell population or multiple cell populations give rise to the BMA lineage [28].

Many stromal cells expressing chemokine (C-X-C motif) ligand 12 (CXCL12) may be precursors of BMAs [33]. Ultrastructural studies suggest that BMAs arise postnatally from anatomically defined CXCL12-positive adventitial reticular (CAR)-like cells [34]. BMAs are derived from one or more stromal progenitor cells that are alkaline phosphatase (ALP)+ and are likely to be in the pericyte position. Paratrabecular and paracortical adipocytes can form their own BMA populations derived from bone-lining cells under certain stress conditions [23]. The formation of BMAs from BM-MSCs has been described for mouse models [35].

The main source of adult WMAs are BM-MSCs, which arise postnatally, express the leptin receptor (Lepr) gene, and settle near vessels (94% of BM-MSCs) [36].

The BM-MSC fatty progenitor hierarchy begins with CD45-CD31-Sca+CD24+ multipotent progenitors that differentiate into WMAs or osteoblasts. At the same time, CD45 and CD31 serve as markers for hematopoietic lineage, while Sca and CD24 serve as markers for adipogenic progenitors. Further differentiation in the adipogenic direction gives rise to CD45-CD31-Sca+CD24 cells, then CD45-CD31-Sca-Zfp234+ preadipocytes, and finally mature BMAs [37]. Other authors have identified Lepr+ BM-MSCs that differentiate into Mpg-rich and Lpl-rich WMA clusters [38].

Single-cell RNA-Seq analysis of BM-MSCs identified nine BM-MSC subpopulations [39]. The earliest mesenchymal progenitors (EMPs) expressed stem cell markers such as Sca-1, Thy, and Cd34. Based on the levels of expression of osteogenic genes, intermediate mesenchymal progenitors (LMPs) were also identified, whose marker may be α-smooth muscle actin (α-SMA). These progenitors (LСPs) are cloned (and then they become Adipoq-Cre) before they differentiate into osteoblasts and BMAs.

In a recent study, mesenchymal progenitor cells were identified before their biclonal differentiation stages, and non-proliferative, adiponectin-expressing LepR [23] progenitor cells of BMAs localized to pericytes were termed medullary adipogenic progenitor cells (MALPs) [39]. MALPs maintain BM vascular homeostasis and promote pathological bone loss in a RANKL-dependent manner [40], and they inhibit bone formation via secreted factors (VEGF, angiopoietin 4 (ANGPT4)) [39]. In addition, MALPs express typical adipocyte markers (i.e., Pparg, Cebpa, Adipoq, Apoe, and Lpl, but not Plin1) and do not contain lipid droplets. However, one study questioned the specificity of the Adipoq-Cre population (described as MALPs) [41].

## 4. Humoral Factors Affecting Adipogenesis and Myelopoiesis

During maturation, BMAs are responsible for the release of adipokines and free fatty acids. Simultaneously, adiponectin secreted by stromal cells during differentiation of BM-MSCs into BMAs stimulates proliferation and multipotency of hematopoietic cells and progenitor cells (HSPCs) via the p38 MAPK pathway [23].

Differentiation of BMAs from BM-MSCs is tightly regulated by transcription factors. Early adipogenesis is associated with CCAAT/enhancer-binding protein CEBPβ/δ transcription factors that activate proliferator-activated receptor gamma (PPARγ) and CCAAT enhancer binding protein A (CEBPα) (whose gene expression levels are increased in rat sBMAs compared with rBMAs) [24]. Osteoblasts and osteocytes have been found to produce factors that can limit or induce BM adipogenesis [42]. Osteocyte-secreted sclerostin (encoded by the SOst gene) promotes expression of the adipogenic transcription factors Ppary and Cebpa in primary human and mouse MSCs in vitro by inhibiting Wnt signaling. Bone morphogenetic proteins can promote adipogenesis by inducing the expression of Ppary and Cebpa [24].

Adipsin released during BMA expansion retroactively affects BM-MSC differentiation and promotes adipogenesis [43]. Human primary BMAs from the femur have been shown to express the pro-osteoclastogenic receptor activator of nuclear factor kappa B ligand (RANKL) [44]. Expression of RANKL is associated with BMA differentiation and with preadipocytes in the BM of aged mice [44,45].

In contrast to WAT, in which leptin is expressed in proportion to adipocyte size, BMAs are characterized by moderate hypertrophy and decreased (unchanged) leptin expression in the presence of positive energy balance [27]. Expression of the stem cell factor gene (kitl) was also detected in WAT and BMAs [46]. Increased adiponectin expression and low Adipoq expression were detected in BMAs compared with WAT [46]. BMAs have high unsaturated lipid contents and lower unsaturated lipid contents, associated with lower bone mineral density [19].

Parathyroid hormone (PTH) regulates the fate of BMCs [47]. Inhibition of PTH signaling in mouse BM stromal cells resulted in an increase in BMAs [47]. Also, lack of expression of PTH1R in BM-MSCs promoted differentiation to adipocytes [47].

Estrogen deficiency is a strong stimulus for BMAT development in rodents and humans. In addition, rBMAs are regulated by other factors, including cold exposure (decrease in rBMAs), fasting leptin [48], exercise [27], and lactation. However, another study reported that BMAs have high basal glucose uptake but lack resistance to insulin, cold exposure, and glucocorticoids [49]. However, clinical studies have shown that insulin increases glucose uptake in BMAs in human thighs [50]. These conflicting results suggest species and local (tibia, femur) differences in BMA metabolism [24]. Accumulation of rBMAs in the proximal tibia is dependent on expression of the gene cavin-1 lipodystrophy (Ptrf) [31]. Positive energy balance and/or the development of obesity, thiazolidiones, glucocorticoids, fibroblast growth factor-21, CD1, and CD2 also contribute to the spread of rBMAs [19].

Recent studies have identified novel subsets of BMA progenitor cells that are recruited to various skeletal and hematopoietic stem cell niches during BM adipogenesis [51]. Long-term hematopoiesis is also regulated by the spatial distribution of BMAs. It has been found that the lipolytic and secretory activity of BMAs can influence the survival and proliferation of hematopoietic cells at different stages of maturation [51].

## 5. Adipocytes as Factors of the Hematopoietic Microenvironment (HIM)

Data on the role of adipocytes as HIM components are rather contradictory. On the one hand, long-term in vitro culture of granulocytic monocytic hematopoietic cells (Dexter culture) decays rapidly in the absence of adipocytes [52]. In parallel, BMAs were also used by [53] as negative regulators of hematopoiesis. With the reduction in the number of BMAs (genetic, pharmacological) in vivo, the recovery of hematopoietic progenitor cells after transplantation improved with a selective expansion of myeloid and granulocytic populations. One possible explanation is competition for space in the BM cavity between BMAs and hematopoietic cells [19,54].

It is suggested that an increase in the amount of BMAs can disrupt the structure of the microenvironment of BM stem cells (BM-MSCs) and their fate. For example, age-related bone loss is associated with a decrease in the proliferative and functional potential of BM-MSCs and an increased ability of BM-MSCs to form adipogenic lineages [55]. Furthermore, the frequency of skeletal stem progenitor cells (SSPCs) decreases with age [56]. BMAs have been shown to be actively produced by pro-inflammatory factors (e.g., IL1α, TNFα, RELA, PPARγ, RANKL, leptin, resistin, chemerin, adipsin, adiponectin, IL1, NRLP3, CCL2/MCP-1, COX2, NFKb, IL1β, TGF-β, CXCL1/2), which negatively affects the number and function of SSPCs [44,57].

In contrast, other work points to the supportive role of adipocytes in hematopoietic cell function in vivo. Foci of ectopic hematopoiesis (myelolipomas) in which adipocytes are closely surrounded by hematopoietic elements have been described [58], frequently found in the adrenal glands [59].

According to electron microscopy studies, BMAs are in direct contact and active interaction with perivascular cells as well as myeloid cells, including central macrophages in erythroblastic islets [34].

Some adipocytes are innervated by the sympathetic nervous system [34]. The data obtained by the authors allowed them to propose pericytes as precursors (progenitors) of adipocytes. By analogy with endothelial niches for hematopoietic cells [60], the existence of a hematopoietic niche of adipocytes has been proposed as a local regulator of the intercellular distribution of energy stored in lipids [34].

HSPCs are in direct contact with BMAs, and conditioned BMA medium promotes HSPC expansion and differentiation ex vivo [34]. BMAs secrete 994 proteins, including positive regulators of HSPC differentiation, motility, and adhesion (e.g., TGFB1, FBLN1, IGFBP2, LGALS1, TIMP1, C3). Of these proteins, 430 are of microvesicular/exosomal origin, highlighting the complex structure of SMA and its paracrine action. However, BMA tissue contains many cell types in addition to BMA including granulocytes, monocytes, and macrophages [61]. Levels of immunoregulatory cytokines were found to be increased in BMAs, including CCL2, CCL5, IL-6, IL-8, IL-10, IL-15, CCR7, CCRL2, and CXCL1 [57]. In addition, increased cholesterol metabolism (1.5-fold increase in free cholesterol) and decreased lipolytic activity [24] were observed in BMAs.

Secretion (and expression) of BMA stem cell factor (SCF or Kit ligand) is essential for the maintenance of hematopoietic cells [62,63]. The limitations of studies on the interaction between BMAs and hematopoiesis are related to the use of irradiation and BM transplantation. At the same time, irradiation causes increased BM adipogenesis in humans [64] and rodents [19,65].

## 6. The Role of Bone Marrow Adipocytes in Myelopoiesis in Obesity

The BMA tissue is considered to be a metabolically active organ that plays a multifaceted role in endocrine function, bone homeostasis, metabolism, and energy conservation [44]. However, BMAs are an understudied aspect of adipocyte biology.

In people with obesity, diabetes, and osteoporosis, a specific site for adipose-tissue-related enlargement of BMAs has been confirmed [66,67].

BMA enlargement in aging/diabetes was found to result in persistent energy storage—a metabolic signature reminiscent of white adipose cells [24].

BM fat cells play an important role in cellular metabolism and hematopoietic lineage [29]. In obese mice, brown adipocyte markers decreased and white adipocyte markers increased (BM) against a background of increased fat storage capacity [68]. It was found that even a short-term high-fat diet (in mice) led to a change in the cellular composition of BM, resulting in adipocyte whitening and activation of invasive Ly6Chigh monocytes in vivo and in vitro [6]; rBMAs undergo hyperplasia, hypertrophy, glycolytic shift (i.e., decrease in mitochondrial oxidative capacity, decrease in mitochondrial membrane potential), and conversion to white adipocytes), and they regulate the processes of myelopoiesis and egress of monocytes from BM [6].

BMAs were found to alter the expression of mitochondrial fusion and division genes in monocytes against a background of obesity in mice. In monocytes from HFD-treated mice, a decrease in the expression of genes regulating mitochondrial fusion (Mfn2, Opa1, Tomm20, Tomm40) was observed, whereas the expression of genes involved in mitochondrial division (Drp1, Ppid, Fis1) was increased [6].

Thus, the accumulation of monocytes in BM precedes an increase in the M1 subpopulation of pro-inflammatory macrophages. White adipocytes caused a shift toward Ly6Chigh, possibly through conversion of Ly6Clow and Ly6Chigh cells, and brown adipocytes maintained Ly6Clow monocyte proliferation. Against a background of increased numbers of circulating Ly6Chigh monocytes, long-term changes were observed in the populations of BM monocyte progenitor cells, indicating some specification (transformation) of the cells. The subsequent increase in the blood monocyte component in obesity may be the result of the combined influence of BM adipocytes, high expression of chemoattractant receptors (CCR2) (as a consequence of a high rate of migration into the tissue), and a response to the depletion of monocytes in the blood [6]. It should be noted that there is a positive correlation between WMA hypertrophy and monocytosis in mice after HFD (3 weeks, 8 weeks). At the same time, VEGFA/B secreted by BMAs can induce proliferation of monocytes. BMAs also stimulate the proliferation of adipocyte progenitor cells and endothelial cells. These breakthrough results suggest that HFD affects hematopoietic myeloid skewing in BM before monocytes invade tissues and promote systemic and peripheral tissue inflammation [6].

Human BMAs have been reported to support the differentiation of CD34+ HSCs (hematopoietic stem cells) into myeloid and lymphoid immune cells [69]. Myelopoiesis was correlated positively with increased adipogenesis and decreased osteoblastogenesis in accelerated aging mouse model 6 (SAMP6). In mice with diet-induced obesity, an increase in BM hematopoietic and lymphopoietic cell populations was correlated with an increase in BM obesity. Lipid-loaded BMAs were associated with the inhibition of HSC growth and differentiation [70]. This suppressive effect was associated with decreased granulocyte-macrophage growth factor (G-CSF) production and increased secretion of neuropillin and lipocalin-2. BMAT is an important source of plasma adiponectin in mice during caloric restriction and in cancer patients receiving radiotherapy or chemotherapy. Adiponectin has a beneficial effect on multipotent stem cell proliferation, but not on sessile progenitor cells, presumably by participating in the maintenance of the HSC pool, underscoring the anti-inflammatory properties of adiponectin [44].

Effects of BMAs on BM-MSCs: BMA expansion is accompanied by the release of pro-inflammatory mediators that have a damaging effect on neighboring cells, induce BM-MSC senescence (via ROS), and decrease the functional properties/number of stem cells [56,57,71]. The results of cytometric analysis showed higher levels of ROS in femoral BMAs compared with WAT from the thigh [57], indicating inhibition of the ability of BM-MSCs to maintain the hematopoietic niche by ROS [72].

Lo et al. showed that under conditions of elevated in vitro glucose levels, β-galactosidase activity and markers of adipogenic differentiation (Pparγ and Fas) were markedly increased, whereas osteogenic markers (Runx2 and Col1a1) were reduced in BM-MSCs, indicating altered potential differentiation [73]. This hyperglycemic state induces inflammation and aging through oxidative-mediated autophagy, ultimately contributing to impaired bone development and hematopoiesis in the bone marrow microenvironment [74]. BMP-2, a detected pro-osteoblastogenic protein, can stimulate bone formation in healthy, non-senescent BM-MSCs. However, in senescent cells, recombinant BMP-2 activates inflammatory, adipogenesis, and cell apoptosis pathways [75]. In mouse models, FOXP1—a regulator of the pro-adipogenic CEBPβ/δ complex in BMAs–has been shown to attenuate aging by downregulating p16 INK4A (encoded by CDKN2A), a cell-cycle repressor that induces G1 phase arrest [76]. Taken together, the BMAs play a critical role in the induction of BM-MSC senescence and, thus, determine the status of the microenvironment in the BM compartment during aging.

Interestingly, the results of a study performed on mice showed a directional differentiation of bone marrow cells into bone marrow macrophages under the influence of macrophage colony-stimulating growth factor (M-CSF) in vitro [77]. At the same time, bone marrow is more dependent on locally secreted M-CSF than on that derived from the periphery. At the same time, Adipoq lineage progenitors have been shown to be important producers of M-CSF and to control the development of macrophage and osteoclast populations in the bone marrow. A unique mechanism of bone marrow homeostasis is proposed, by which the bone marrow microenvironment regulates the separation of osteoclast formation and monocytopoiesis from the systemic regulation of tissue-resident macrophages [78].

## 7. Additional Factors Affecting Myelopoiesis in Obesity

Of interest is the role of the mannoselectin receptor (Mrc1) in the activation of myelopoiesis under dysmetabolic conditions associated with pro-inflammatory markers (and cells) in the blood [79]. Mrc1 deficiency is associated with a decrease in circulating neutrophils and pro-inflammatory CCR2+ monocytes and low infiltration of these cells into tissues, against a background of increased presence of adipocytes in the BM in obese mice (Mrc1-/-) [80] (slowing the development of obesity) [79].

Experimentally (in mice), the role of sex hormones in the development of obesity, the formation of myeloid colonies in the BM, and the inflammation of adipose tissue has been demonstrated. Thus, androgens significantly enhance myelopoiesis in BM, and estrogens (e.g., myeloid estrogen receptor alpha) have a protective effect [81].

In addition, circulating monocytes respond to dietary lipids and chemokines, produce cytokines themselves, and give rise to tissue macrophages in obesity. Stimulation of myeloid cells in obesity has been shown to occur under the influence of saturated lipid palmitate (PA) and the chemokine monocyte chemoattractant protein 1 (MCP1) [82].

Cholesterol metabolism plays a critical role in regulating the proliferation of hematopoietic stem and progenitor cells [83]. Hypercholesterolemia increases the number of circulating monocytes by stimulating the proliferation of hematopoietic stem and progenitor cells in the BM and spleen [81]. Classical monocytes exit the BM in a CCR2-dependent manner. CCL2 and CCL7 are ligands for CCR2 and contribute to the maintenance of stable concentrations of circulating monocytes [84].

The spleen is the main site of extramedullary monocytopoiesis and contains a reserve of over one million monocytes. A general population of monocytes has been found to exist in the BM and in the spleen to generate monocytes and monocyte-derived macrophages [84].

## 8. Monocytes

### 8.1. Characteristics of Monocytes in Metabolic Syndrome (Obesity)

Monocytes are central cellular elements of the innate immune response and are involved in homeostasis, immune defense, and tissue repair [85].

Monocytes are divided into classical (CM, CD14++CD16-−) [4,6], non-classical (NCM, CD14dimCD16+) (or CD14+CD16++ [84]), and intermediate (IM, CD14+CD16+ HLA-DR+CD86+CD11c+) (and CD14++CD16+) [86]. Classical and intermediate populations of monocytes are sensitive to CCL2, CCL3, and CCL4, while non-classical monocytes are sensitive to CX3CL1 [85,87].

The surface marker of classical monocytes is the lipopolysaccharide (LPS)-binding CD14 receptor, whereas the FcγRIII (CD16) receptor is not expressed [84]. The fate of classical monocytes is regulated by the sequential action of the transcription factors PU.1 (Sfpi1), Irf8, and Klf4 [88,89].

Non-classical monocytes develop from classical monocytes under the control of Nr4a1 (TR3/Nur77) [88,89]. Non-classical monocytes have been shown to be “proactive” inflammatory cells and have lower expression of CD11b (integrin alpha M or complement type 3 receptor) and CD36 [4]. Expression of these surface molecules in peripheral blood monocytes has been used to characterize the inflammatory response in metabolic disorders (i.e., diabetes and obesity) [4]. In addition, a surface marker—human leukocyte antigen (HLA)—is located on the surface of non-classical monocytes. An expression level of HLA-DR of less than 80% indicates immunosuppression, and when this indicator falls to a threshold of less than 30%, it indicates immunoparalysis [84].

The intermediate monocyte population is characterized by a transcriptional profile: LYZ, S100A8, CD14, S100A10, HLA-DRA, CD74, IFI30, HLA-DPB1, CPV, and 6-sulfoLacNAc expression [90]. CD14+CD16+HLA-DR+CD86+CD11c+ cells showed higher expression of class II molecules and IL12 production, comparable ATP production and phagocytic potential, but lower adhesiveness compared with classical monocytes [85]. They are able to generate ROS and pro-inflammatory cytokines (IL1β and TNFα) in response to LPS. Intermediate monocytes have a high proangiogenic capacity and are efficient in processing and antigen presentation, with strong HLA-DR and Toll-like receptor expression [91,92].

It was found that, in humans, intermediate and non-classical monocyte populations emerge from the pool of classical monocytes [93]. Mathematical modeling showed a linear trajectory from classical monocytes (lifespan in blood: 1–2 days) to non-classical monocytes, which does not exclude other pathways of monocyte differentiation, including outside the blood circulation [94]. The effect of endotoxin resulted in rapid loss of all monocyte subpopulations. Subsequent monocyte recovery began with the formation of a pool of classical monocytes, followed by intermediate and non-classical monocytes [85]. It has been previously established that non-classical monocytes are the primary inflammatory monocytes in acute and chronic inflammation [4].

Reprogramming of monocyte precursors in the bone marrow has been shown to occur during infection [85].

Classical monocytes are primed for phagocytosis, innate sensory/immunological responses, and migration; intermediate monocytes differ in terms of CCR5 expression and are capable of presenting antigens, secreting cytokines, and regulating apoptosis and differentiation; non-classical monocytes are involved in adhesion and complement-mediated and Fc-gamma phagocytosis. At the same time, a large heterogeneity is observed between the three major populations of monocytes, mediated by differential expression of transcription factors [85].

Monocytes differ in size, granularity, and nuclear morphology. Monocytes express various chemokine receptors and adhesion molecules. This also mediates the heterogeneity of the tissue macrophages that they produce [95].

### 8.2. Activation of Myelopoiesis in Bone Marrow

Elevated HDL cholesterol levels play an anti-atherogenic role based on suppression of myeloid proliferation in bone marrow. In obesity, inflamed adipose tissue increases the proliferation of hematopoietic BM cells, leading to exacerbation of inflammation and associated pathological processes [96]. Hyperglycemia and diabetes are associated with increased production of inflammatory myeloid cells in the BM, which exacerbates complications associated with diabetes mellitus, including atherosclerosis [97].

BM activation is associated with metabolic syndrome (MetS) and its individual components: an increased number of lymphocytes and a systemic inflammatory response. Studies in mice have shown that low levels of high-density lipoprotein (HDL) cholesterol and hypercholesterolemia are associated with increased myelopoietic BM activity, leading to increased neutrophilia and monocytosis [96].

Dietary habits influence the number and composition of the three circulating monocyte populations. Obesity induces monocytosis of intermediate and non-classical populations against a background of increased TLR4/8 expression and secretion of pro-inflammatory cytokines (e.g., IL1β, TNF) in response to LPS or ssRNA stimulation. [85]. At the same time, the association between BM activation, increased hematopoiesis (i.e., release of hematopoietic progenitor cells into the bloodstream), and MetS persists even in the absence of systemic inflammation and is an early phenomenon that occurs in response to MetS [96]. In addition, morbid obesity in MetS is associated with a meta-inflammatory response and the development of cardiovascular complications—specifically, with the transition from inflammatory classical CD14+CD16- monocytes to anti-inflammatory non-classical CD14dimCD16+ monocytes. A study of the profile of immune cells in the peripheral blood of patients with morbid obesity showed an increase in the number of senescent CD14dim monocytes; however, the association between monocytes and morbid obesity was observed, but not with MetS [98]. It is suggested that monocyte aging may be reversible [99].

Body mass index (BMI), waist circumference, and body fat percentage have been shown to be directly related to an increase in the proportion of non-classical monocytes and a decrease in the number of classical monocytes. This relationship was observed against a background of overweight and obesity. A decrease in the proportion of classical monocytes and a significant increase in the non-classical monocyte subpopulation were also associated with HDL levels (and showed no association with obesity-related anthropometric parameters). HDL is a blood plasma protein that can bind to lipid molecules (e.g., triglycerides, cholesterol) and is involved in cholesterol excretion. Elevated HDL levels are thought to limit the proportion of inflammatory non-classical monocytes and promote an increase in the population of classical monocytes, which do not have a marked inflammatory potential. Also, when primary human monocytes were cultured under high/low-HDL conditions and LPS stimulation, a percentage increase in non-classical monocytes and a decrease in classical monocytes were observed [100].

In obesity, circulating monocytes express high levels of CX3CR1, indicating increased chemotactic potential toward CX3CL1 secreted by adipocytes, as evidenced by the high numbers of monocyte-derived macrophages in adipose tissue [101].

Short-term fasting has been found to reduce the number of all populations of monocytes in healthy individuals [85,102]. Similar results were obtained in a study of fractionated (and time-limited) low-fat diets in obese mice (male C57BL/6) [3].

### 8.3. Monocytes in Peripheral Blood in Metabolic Syndrome (MS)

Central obesity is associated with a chronic inflammatory state and sympathetic nervous system hyperactivity [4]. Catecholamines, epinephrine, and norepinephrine are potent modulators of neuroendocrine, immunological, and inflammatory networks that regulate the inflammatory response of monocytes under normal and pathological conditions. In obesity, WAT is infiltrated by large numbers of peripheral blood monocytes [4].

Severe obesity is characterized by higher numbers of peripheral blood monocytes, a relative increase in CD16+ monocyte subpopulations, and increased levels of surface markers of inflammation in all populations compared with a lean physique [103].

CD16+ monocytes are thought to be present in the marginal pool and their mobilization and proliferation are associated with obesity (under stress conditions) [4]. Expansion and accumulation of adipose tissue is partially associated with proliferation and an increase in the number of circulating pro-inflammatory monocytes [104].

One indicator of chronic monocyte activation in obesity is translocation of the NFkB nucleus and increased expression of pro-inflammatory cytokines. Studies have shown that the severity of obesity or components of metabolic syndrome is associated with an increase in CD16+ monocytes (IM, NCM) [104,105,106,107]. Another study found a significant negative relationship between IM monocyte count and waist circumference in a cohort of obese patients with low variance in BMI [108]. Strong associations were found between total monocyte count, sCD163 (particularly in metabolic syndrome, [109]), and IM and systemic macrophage activation score. The relationship between the amounts of IM and sCD163 may indicate a general systemic activation of the monocyte–macrophage junction of the innate immune system in people with obesity. This could also be related to the monocyte mobilization chemokine CCL2, which increases in obesity [102,108].

It was found that two heterogeneous subpopulations of intermediate monocytes were identified in the peripheral blood of obese humans (and healthy donors), based on high and intermediate expression, respectively, of the surface marker HLA-DR (DRmid, DRhi). These cells differentially expressed membrane proteins such as CD62L, CD11a, CX3CR1, and CCR. At the same time, the phenotypic profile of DRmid resembled that of classical monocytes, whereas that of DRhi resembled that of non-classical monocytes. However, DRmid was characterized by weak migratory activity after CCR2 ligation and adhered to TNF-activated endothelium. In obese humans, the proportions and absolute amounts of DRmid in peripheral blood were increased [110].

Expression of the surface markers CD14, CD16, CD36, CD45, and CD64 decreased against a background of weight loss. In obesity, high expression of the monocyte activation marker CD300e was observed in peripheral blood, which decreased significantly in the initial phase of weight loss but increased in the subsequent phase of body weight maintenance [108].

### 8.4. Monocytes/Macrophages in Adipose Tissue in Metabolic Syndrome

**Figure 2:**
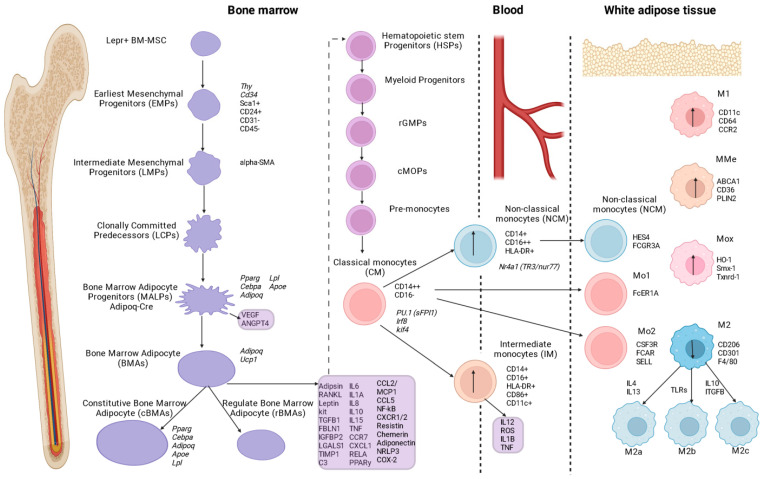
The scheme of adipocyte development and maturation in bone marrow and its influence on monocyte and macrophage branching in metabolic syndrome (obesity).

A study of the cellular composition of WAT in obesity revealed three populations of monocytes, including non-classical monocytes (FCGR3A, HES4) and classical monocytes: Mo-1 (FCER1A) and Mo-2 (CSF3R, FCAR, SELL) [111] (Figure 2).

At the same time, obesity is associated with a high dynamic macrophage population in adipose tissue (AT) of 40–60% (compared to the thin population, where this indicator was 10–15% AT), characterized by a pro-inflammatory potential. The traditional classification of macrophages based on functional properties includes a pool of M1 pro-inflammatory cells (surface markers F4/80, CD11c, iNOS) and M2 anti-inflammatory cells (secretion of IL4, IL10, IL13, and the surface markers CD206, CD301, CD68, CD11b, arginase 1, and F4/80). In addition, AT macrophages (ATMs) are divided into resident and recruited macrophages, which are formed from monocytes [1]. Human ATMs in adipose tissue are thought to exhibit a mixed phenotype in obesity, characterized by the common expression of CD11c (M1), CD206, and CD163 (M2). At the same time, macrophage CD11c+ and CD206+ are correlated with insulin resistance, and the number of CD11c+ and CD163+ cells is associated with BMI. In addition, CD163 can be tracked with HOMA-IR [112].

Macrophages derived from BM-M0 form M1 or M2 macrophage subtypes, depending on the microenvironment and mediators. Moreover, M2 macrophages differentiate into M2a (under the influence of IL4 and IL13), M2b (stimulation of TLRs), and M2c (under the influence of IL10 and TGFβ) [101].

Metabolically activated macrophages and oxidized macrophages are involved in the development of insulin resistance associated with obesity [113]. MMEs have pro/anti-inflammatory properties and are triggered by saturated fat or high insulin levels. Moss is induced by oxidized phospholipids [114].

CCR2/Ly6Chi monocytes were found to invade and proliferate in adipose tissue and/or differentiate into macrophages. At the same time, surface expression of the CD11c marker on macrophages might indicate that they originate from peripheral blood monocytes. However, this receptor was also found on dendritic cells in adipose tissue [112].

The results of a study (in mice and humans) using single-cell RNA sequencing identified three subpopulations of adipose tissue macrophages (ATMs), tetraspanin CD9, and Ly6C as markers, thereby classifying the macrophages as Ly6C+ (monocytes), Ly6C-CD9-, and Ly6C-CD9+ [115]. Ly6C+ monocytes were evenly distributed in tissues outside the CLS and were adipogenic. CD9+ ATMs were located within the CLS, had a large amount of intracellular lipids, and expressed pro-inflammatory genes. Lipid-loaded CD9+ ATMs localized in the CLS were also found in the adipose tissue of obese patients [115,116]. CD9+ ATMs were designated lipid-associated macrophages (LAMs) and expressed high levels of the lipid receptor Trem2, whose knockout in BM exacerbated the metabolic consequences of obesity. This suggests that macrophages in adipose tissue also have beneficial functions (including reparative functions) in obesity against a background of pro-inflammatory potential [112]. The Trem2+ subgroup of ATMs has been shown to prevent adipocyte hypertrophy, inflammation, and metabolic dysfunction [116].

Neuronal-associated macrophages (SAMs) promote Slc6a2 receptor-mediated norepinephrine clearance and subsequently degrade it through the enzymatic action of monoamine oxidase A. Activation of the sympathetic nervous system contributes to the pro-inflammatory phenotype of SAMs through the excessive accumulation of norepinephrine [117].

### 8.5. Cells Formed from Bone Marrow Monocytes: Main Players in the Pathogenesis of Metabolic Syndrome

Some obesity-related alterations in the immunological component of adipose tissue have been found to persist after weight loss, suggesting complex mechanisms of adipose tissue dysfunction and disruption of metabolic homeostasis at the system level [118].

For example, altered distribution of the immune system’s monocyte–macrophage connection has been found in brain structures.

The hypothalamus is an important part of the central nervous system and regulates systemic energy and glucose metabolism. Neurons of the nucleus arcuatus of the hypothalamus (ARC) and the area near the median eminence (ME) do not have a blood–brain barrier, but they sense peripheral metabolic signals (leptin, insulin) to maintain metabolic homeostasis. Obesity leads to inflammation in the ARC, which is maintained by microglia (immune cells) and promotes disease progression [119]. ARC microglia are readily activated in response to short-term exposure to HFD and trigger an inflammatory response to saturated fatty acids in the hypothalamus. Simultaneously, BM macrophages are also present in the hypothalamus [120]. However, a study in mice showed that circulating myeloid LysM GFP cells are not actively recruited to the hypothalamus ARC even during chronic HFD, which was unexpected due to the permeability of the ARC vasculature after early HFD feeding [121]. However, another study demonstrated the presence (approximately 30%) of CD68+ myeloid cells of peripheral origin expressing GFP in ARC [122]. The ability of hypothalamic macrophages to proliferate in response to HFD feeding (in mice) has also been demonstrated. Moreover, most hypothalamic LysM GFP cells originate from the BM and colonize the hypothalamus during postnatal lactation, when hematopoiesis shifts from the liver to BM [121].

Inhibition of hypothalamic fractalkine (CX3CL1) reduces diet-induced hypothalamic inflammation and recruitment of BM monocytic cells to the hypothalamus, reduces obesity, and protects against glucose intolerance [123].

## 9. Conclusions

Obesity is associated with changes in the qualitative and quantitative components of BMAs. At the molecular level, this affects the process of normal hematopoiesis [19,44,63,69].

Monocytes play a key role in the development of metabolic syndrome, and particularly obesity. Given the complex features of their development from progenitor cells, whose regulation is mediated by their interactions with bone marrow adipocytes, the importance of a detailed study of the heterogeneous composition of monocytes at the molecular and systemic levels becomes apparent. Modern research aims to find evidence to support the hypothesis of a primary alteration in the adipocyte component of the bone marrow [11] against a background of obesity that induces monocytosis and further migration of monocytes to adipose tissue and other organs, followed by their differentiation into macrophages and the development of systemic, sluggish inflammation.

Interestingly, monocytes are CNDP2+ cells (i.e., macrophages, immune and epithelial cells), where biosynthesis of N-lactoylphenylalanine (Lac-Phe) (from lactate and phenylalanine) occurs against a background of physical activity, which has the function of suppressing appetite and reducing obesity. [124]. Studies in middle-aged men with central obesity have shown a direct association between physical activity and a limitation of pro-inflammatory monocyte migration and a decrease in their adhesive properties [125]. In addition, it has been detailed how physical activity can be used to combat obesity-induced bone marrow dysfunction [126].

A high-fat diet has been shown to induce a TNF-dependent increase in circulating inflammatory monocytes that predicts increased blood insulin levels and insulin resistance [127]. Therefore, pro-inflammatory mediators secreted by adipose tissue could be potential targets to reduce the process of monocytopoiesis/monocytosis.

A promising direction in anti-obesity therapy is the development of new techniques using miRNAs. Manipulation of human THP-1 macrophages exposed to IL-4 showed a change in their phenotype toward anti-inflammation, while their energy metabolism was reprogrammed by increasing miRNA-21/99a/146b/378a and decreasing miRNA-33. Intraperitoneal administration of THP-1-IL4-exo to obese wild-type mice promoted lipophagy, mitochondrial activity, and 3T3-L1 fat cell accumulation. Myelopoiesis was also reduced due to reprogramming of inflammatory signals and metabolism of circulating Ly6Chi monocytes [128].

These data argue for monocytes as indicators of changes in the body’s metabolism and the possibility of developing therapeutic strategies to combat obesity and components of metabolic syndrome based on manipulations of the monocyte connection in the immune response. Thus, an in-depth study of the heterogeneity of bone-marrow-derived monocytes and adipocytes could provide answers to many questions about the pathogenesis of obesity and reveal their therapeutic potential.

## Data Availability

No new data were created or analyzed in this study. Data sharing is not applicable to this article.
